# Five-year outcomes of trauma-specific function in patients after acute blunt popliteal artery injury: a matched cohort analysis

**DOI:** 10.1186/s13018-022-03145-x

**Published:** 2022-05-07

**Authors:** Gang Liu, Jialei Chen, Zhou Xiang

**Affiliations:** Department of Orthopedics, Orthopedic Research Institute, West China Hospital, West China Medical School, Sichuan University, #37 Guoxue Road, Chengdu, 610041 Sichuan Province People’s Republic of China

**Keywords:** Popliteal artery injury, Blunt trauma, Function, Risk factor, Long-term follow-up

## Abstract

**Background:**

Few studies focus on the trauma-specific functional outcomes after surgical revascularization and risk factors contributing to poor outcomes in patients with acute blunt popliteal artery injury (PAI). The objective of this study was to investigate the long-term trauma-specific functional outcomes in patients with acute blunt PAI and identify the associated risk factors.

**Methods:**

There were 36 patients with acute blunt PAI who require surgical revascularization at a national trauma center of West China Hospital of Sichuan University between March 2010 and April 2019. After propensity matching, each patient was matched to one patient who did not have a concomitant vascular injury in control cohort. Functional outcomes were assessed with trauma-specific functional scores, physical examination of range of motion, nerve functional status and knee stability. A logistics regression model was established to determine the independent risk factors.

**Results:**

The 5-year (range 2–10 years) follow-up showed that 22 patients (22/36, 61.1%) had functional deficit due to limited activity or chronic neurological symptoms. Patients in vascular cohort had significantly decreased FIM score and AHFS score compared with matched patients without vascular involvement (*P* = 0.003 and *P* < 0.001), whereas there was no statistically significant difference in KSS (*P* = 0.136). Spearman correlation analysis of functional scores in vascular cohort showed that the FIM score was positively correlated with AHFS score (*r* = 0.926, *P* < 0.001), but not correlated with the KSS (*r* =  − 0.007, *P* = 0.967). Additionally, there was significant difference in the range of motion of ankle between two groups (*P* < 0.001 and *P* = 0.034). Logistic regression analysis further demonstrated nerve injuries and compartment syndrome were risk factors for poor ankle function after surgery (OR 22.580, *P* = 0.036 and OR 12.674, *P* = 0.041).

**Conclusion:**

Most patients who sustained blunt PAI had significant functional deficit associated with limited activity and chronic neurological symptoms of ankle and foot, and poor functional outcomes were related to nerve injury and compartment syndrome. Therefore, early and effective decompression for compartment syndrome remains the only potentially modifiable risk factor for improving functional outcomes following PAI.

## Background

Acute popliteal artery injury (PAI) is a potentially limb-threatening medical emergency associated with fracture/dislocation of knee [1, 2]. Previous investigations have demonstrated that 56.8%-61% of the injuries were involved in blunt mechanism. Compared with penetrating trauma, blunt trauma is thought to yield high amputation rate and poor clinical outcomes because of complicated soft tissue injuries [3, 4]. Non-operative treatment is definitely not recommended if popliteal artery injuries are significant and present with acutely ischemic limbs [5]. Although significantly reduced amputation rates for PAI have been achieved with improved revascularization technology, it is still a challenge for orthopedic surgeons mainly due to the heterogeneous patient populations with moderately to severely blunt PAI [6, 7].

Despite extensive reviews on limb salvage and the difference between blunt and penetrating PAI [3, 4, 8], minimal studies have focused on the functional outcomes after limb salvage in this patient population. Additionally, the major limitations of present studies which had explored functional outcomes were the focus of self-care disability and activities of daily living instead of trauma-specific function, and the unknown reason for functional loss following treatment of PAI [3, 9]. A detailed description of the long-term functional outcomes from trauma-specific design for patients with acute blunt PAI and risk factors contributing to poor trauma-specific function is lacking. Therefore, the purpose of this study was to evaluate trauma-specific functional outcomes in patients with acute blunt PAI after successful limb salvage. The research also identified risk factors contributing to poor functional outcomes.

## Methods

### Inclusion and exclusion criteria

Patients were identified through a search of prospectively gathered database of patients with acute blunt PAI who require surgical revascularization at a national trauma center of West China Hospital of Sichuan University between March 2010 and April 2019. Inclusion criterion was patients who had acute blunt PAI requiring surgical revascularization. Patients were excluded if they were followed for < 2 years, sustained PAI caused by non-trauma or penetrating injury, or were treated non-operatively. Patients who had undergone primary surgery before being admitted to our hospital were also excluded. Additionally, all patients with limb salvage should receive physiotherapy by a professional specialist and be followed up in our outpatient interviews for a minimum of 2 year after surgery.

**Fig. 1:**
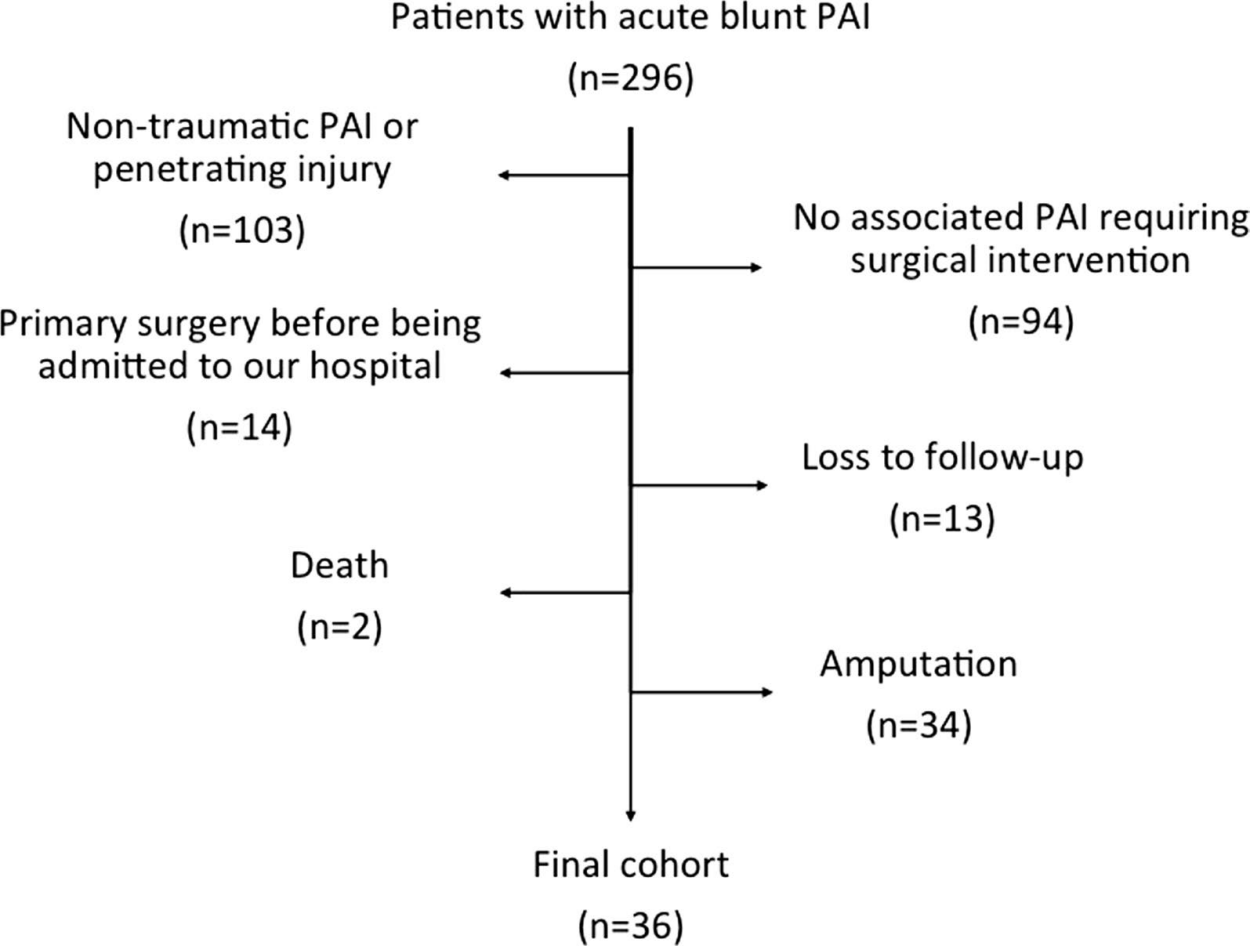
Flow diagram depicting patients arrived at final vascular cohort

A total of 296 patients with acute blunt popliteal artery injury were admitted to the institution. Of these patients, limb salvage was obtained in 36 patients after surgical procedures, and these patients were identified in vascular cohort (Fig. 1). In order to compare limb function during the follow-up, each patient was matched to one patient who did not have a vascular injury in control cohort. The matching was based on age at injury (within 5 years if the age was < 50 years and within 10 years if the age was ≥ 50 years), knee dislocation (KD) grade [10] and knee fracture classification [11]. According to the matching system, 14 patients in each cohort had knee dislocation, including 5 KD-III L, 6 KD-III M, 2 KD-IV and 1 KD-V. A total of 22 patients with knee fracture in each cohort, including 5 patients with extraarticular fracture, 9 with partial articular fracture and 8 with complete articular fracture (Table 1).

**Table 1:** Demographic data and injury details in vascular and control cohorts

	Vascular injury (n = 36)	No vascular injury (n = 36)	*P* value
Age (year)	41.5 (16–72)	42 (21–74)	0.057
Sex (male/female) (no.)	31/5	28/11	0.089
Follow-up (year)	5 (2–10)	4 (2.5–7)	0.073
Injury Severity Score (points)	11 (9–22)	4 (4–13)	< 0.001
Mechanism of injury, no. (%)			0.525
Fall	9 (25%)	13 (36.1%)	
Crush	10 (27.8%)	7 (19.4%)	
Traffic accident	17 (47.2%)	16 (44.4%)	
Knee dislocation (KD) grade, no. (%)			–
I	0	0	
II	0	0	
III L	5	5	
III M	6	6	
IV	2	2	
V	1	1	
Knee fracture classification, no. (%)			–
Extraarticular	5	5	
Partial articular	9	9	
Complete articular	8	8	
Nerve injuries, no. (%)	25 (69.4%)	6 (16.7%)	< 0.001
Compartment syndrome, no. (%)	16 (44.4%)	5 (13.9%)	0.004

The values are given as the medians with the range or counts with percentages in parentheses

### Postoperative management

Patients had delayed passive activity of knee joint at least 3 weeks after surgery with the assistance of an external fixator, progressive active and weight-bearing exercise at least 6 weeks and 12 weeks after surgery, respectively. However, if patients had nerve or complicated soft tissue injuries, weight-bearing exercise should be postponed to 6 months after surgery. Early active and passive activity of ankle joint was recommended.

### Data collection

A prespecified form was used to general data from patients' medical records and outpatient interviews, including the Injury Severity Score (ISS) [12], associated injuries, surgical interventions and functional outcomes at the follow-up period. Ischemia time was defined as the time interval from trauma to blood flow restoration. Complicated soft tissue injury was defined as open fracture and/or dislocation around knee. Nerve injury was diagnosed according to clinical symptom and electromyogram. The diagnosis of compartment syndrome was based on clinically apparent compartment hypertension or after intracompartmental pressure measurement.

### Functional assessment

Modified Functional Independence Measure (FIM) score was used to evaluate the degree of dysfunction of lower extremity from three aspects, including pain, locomotion and climb stairs. The patient-reported functional score for each aspect ranges from 1 (full dependence on assistance) to 4 (full independence), giving a maximum total score of 12 representing full independence if three aspects are added together (Table 2). Patients were considered to have excellent or good result if FIM score was ≥ 10, fair 6 to 9 points and poor < 6 points. Additionally, Knee Society Score (KSS) and Ankle–Hindfoot Scale from American Orthopaedic Foot and Ankle Society (AHFS, short for AOFAS-AHFS) were assessed, and KSS < 69 points or AHFS score < 75 points were defined as poor functional results [13, 14]. The ranges of motion and nerve status were also assessed with physical examination at the end of follow-up visit. If the patient had a knee dislocation, with or without knee fracture, physical examination tests of knee stability included the Lachman test, valgus stress test, varus stress test and external rotation drawer test.

**Table 2:** Modified Functional Independence Measure (FIM) score of lower extremity

Index	FIM score			
	1 point (dependent, total help required)	2 points (dependent, partial help required)	3 points (independent, with device)	4 points (independent, without assistance)
Pain	Severe	Moderate	Slight	Painless
Locomotion	Walking < 15 m requiring help > 1 person	Walking < 45 m requiring standby supervision	Walking > 45 m with brace or crutch	Walking > 45 m without assistance
Climb stairs	Cannot climb stairs	Can only climb up stairs with handrails	Can climb stairs with handrails	Unlimited climbing stairs

### Statistical analysis

Continuous variables are expressed as medians and interquartile range (IQR) and analyzed by Mann–Whitney U test because of the nonparametric nature of the data and associated small sample size. Categorical variables were shown as frequencies (percentages), and compared by *χ*^2^ or the Fisher exact test. Spearman correlation analysis was performed to assess whether FIM score was associated with KSS or AHFS score. A multivariate logistics regression model was established, and an enter method was utilized to determine independent risk factors for poor functional outcomes. Statistical analyses were performed using SPSS 25.0 software (IBM Corp, Armonk, NY). All statistical tests were a two-tailed, and significance was set at *P* < 0.05.

## Results

### Patient characteristics

This study consisted of 31 males (86.1%) and 5 females (13.9%). The median age was 41.5 years (range 16–72 years). The median follow-up time was 5 years (range 2–10 years). The average ISS for patients with complicated soft tissue injuries in this study was 13.5 points (range 13–22 points), and none of the patients had an ISS > 25. All surgical procedures were performed with open repair, including Fogarty thrombectomy in 4 patients (11.1%), direct repair in 9 (25.0%) and autogenous vein grafts in 23 (63.9%). Flow-restored arterial repair was obtained ≥ 8 h after injury in all patients with an average of 22.9 h (range 8 to 61 h). Of these patients, delayed repair < 10 h were found in 3 patients, 10–15 h in 6 patients, 16–24 h in 16 patients, > 24 h in 11 patients. External fixators were used to stabilize the knee joint before vascular repair in 23 patients (63.9%). Fasciotomy was performed in 19 patients (52.8%) as a prophylactic procedure or due to a clinically apparent compartment syndrome. The technique we used was a two-incision four-compartment fasciotomy.

### Functional outcomes

The functional outcomes of lower extremity are presented in Table 3. Using the FIM score to quantify the level of dysfunction of lower extremity of patients with successful limb salvage, we found moderate-to-severe degree of impairment, with an overall outcome score of 9.0 (4–12). The median of KSS score was 83.5 points (range 74 to 100 points), rated as excellent or good in all patients (100%). The median of AHFS score was 68 points (range 24 to 99 points), rated as excellent or good for 16 patients (44.4%) and poor for 20 patients (55.6%). Patients in vascular cohort had significantly decreased FIM score and AHFS score compared with matched patients without vascular involvement (*P* = 0.003 and *P* < 0.001), whereas there was no statistically significant difference in KSS (*P* = 0.136).

**Table 3:** Functional outcomes of lower extremity in vascular and control cohorts

	Vascular injury (n = 36)	No vascular injury (n = 36)	*P* Value
Functional scores (points)			
FIM	9.0 (4–12)	10.5 (5–12)	0.003
KSS	83.5 (74–100)	92 (75–100)	0.136
AHFS	68 (24–99)	100 (90–100)	< 0.001
Range of motion (°)			
Extension of knee	3.5 (0–10)	5 (0–9)	0.865
Flexion of knee	115 (80–145)	120 (100–145)	0.083
Dorsiflexion of ankle	− 10 (− 30–45)	45 (35–45)	< 0.001
Plantar flexion of ankle	40 (25–50)	45 (40–45)	0.034

The values are given as the medians with the range in parentheses

*FIM* Functional Independence Measure; *KSS* Knee Society Score; and *AHFS* Ankle–Hindfoot Scale

On physical examination, there were no significant differences the final ranges of motion of knee between two cohorts (*P* = 0.865 and *P* = 0.083). However, there were significant differences in activity of dorsiflexion and plantar flexion of ankle (*P* < 0.001 and *P* = 0.034) (Table 3). There were also no significant differences in the results of physical examination tests of knee stability, including the Lachman test, valgus stress test at 0°or 30°, varus stress test at 0°or 30° and external rotation drawer test. Of these 25 patients who had initial nerve injuries, 14 regained normal nerve function and 11 had chronic neurological symptoms at the time of final follow-up. The 5-year clinical follow-up showed that 22 (22/36, 61.1%) patients with FIM ≤ 9 were recorded as having functional deficit after surgery because of limited activity or chronic neurological symptoms.

### Potential risk factors for poor functional outcomes

**Fig. 2:**
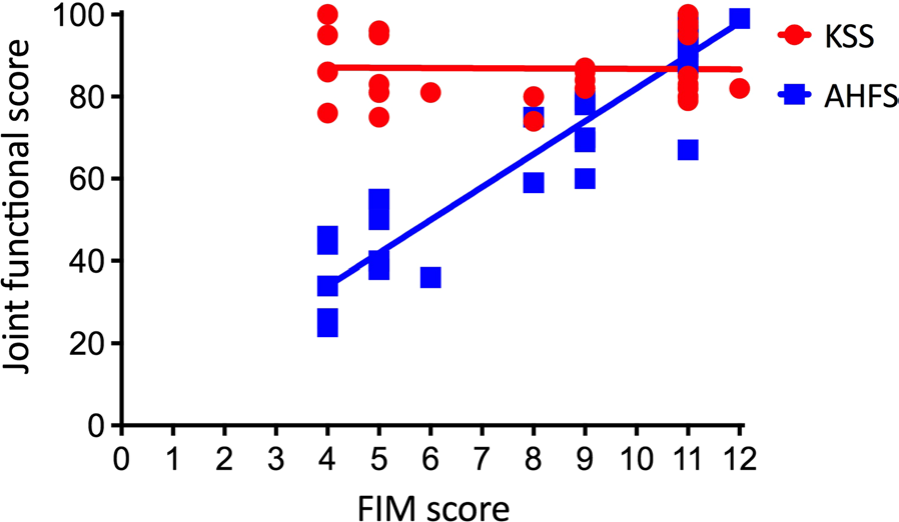
Correlation analysis of FIM score with joint functional score in vascular cohort. *FIM* Functional Independence Measure; *KSS* Knee Society Score, functional score of knee; *AHFS* Ankle–Hindfoot Scale, functional score of ankle

Spearman correlation analysis of functional scores in vascular cohort showed that the FIM score was positively correlated with AHFS score (*r* = 0.926, *P* < 0.001), but not correlated with the KSS (*r* =  − 0.007, *P* = 0.967) (Fig. 2). Logistic regression analysis further demonstrated nerve injuries and compartment syndrome were risk factors for poor ankle function after surgery (OR 22.580, *P* = 0.036 and OR 12.674, *P* = 0.041) (Table 4).

**Table 4:** Potential risk factors for poor functional outcomes

Variable	Odds ratio (95% CI)	*P* value
ISS	0.688 (0.289–1.641)	0.399
Complicated soft tissue injury	1.294 (0.047–35.430)	0.879
Nerve injuries	22.580 (1.228–415.024)	0.036
Compartment syndrome	12.674 (1.116–143.936)	0.041
Duration of ischemia	0.955 (0.870–1.048)	0.331

*ISS* Injury Severity Score

### Complication after surgical revascularization

Observed complications after surgery included shock in 7 patients, deep infection in 5 patients and gastrocnemius necrosis in 25 patients. Compartment syndrome, which is the most severe potential complication after revascularization, was not observed in this series. Repeated debridement was required in 21 patients due to severe soft tissue damage and infection, with an average hospital stay of 21.7 days (range 3–47 days).

## Discussion

To our knowledge, this is the first trauma-specific study investigating the long-term functional outcomes in patients with acute blunt PAI after surgery. The 5-year clinical results of our study suggested that most patients who sustained blunt PAI had significant functional deficit associated with limited activity and chronic neurological symptoms of ankle and foot. Additionally, we also found that long-term outcomes were related to nerve injury and compartment syndrome. Early and effective decompression for compartment syndrome remains the only potential modifiable risk factor for improving functional outcomes following PAI.

Few studies reported trauma-specific functional outcomes for patients with blunt PAI after revascularization. We are aware that only two studies gave a detailed account of self-care disability or daily activity, although they did not include trauma-specific functional outcomes [3, 9]. An analysis of 64 patients with traumatic PAI at 1-year clinical follow-up showed that 30 patients returned to their normal activity level [3]. Similarly, 12 of the 61 survivors with acute blunt PAI had a basic mobility score that fell within the normal range and 15 patients had a normal measure of daily activity at the median follow-up of 11.2 years in another study [9]. Our study confirmed these findings and further explored that the poor subjective function of lower extremity was mainly caused by dysfunction of ankle and foot due to limited activity and residual neurological symptoms. It may be related to complicated soft tissue injuries, prolonged limb ischemia, nerve injuries and compartment syndrome. In addition, nearly two-thirds of the patients had used external fixators for prolonged immobilization because of complicated soft tissue injuries in our series. Despite this, there was no significant difference in postoperative functional scores and range of motion of knee between the two groups.

Compartment syndrome was associated with persistent sequelae after traumatic PAI, particularly presented as long-term sequelae of foot and ankle [15, 16]. A retrospective series of 60 patients who underwent fasciotomy for acute compartment syndrome following limb trauma reported 42 patients had persistent sequelae including motor weakness, paresthesia and dysesthesia [17]. A similar series demonstrated that deformity and functional impairment in the foot and ankle secondary to ischemia are determined by fibrotic cicatrix [18]. Therefore, persistent sequelae were associated with a higher rate of reoperation, post-fasciotomy complications and neuromuscular dysfunction. It may be mainly related to prolonged ischemia because of delayed diagnosis and treatment [19, 20]. In our series, fasciotomy cannot be performed within ≤ 6 h since the majority of patients (27/36) were treated after 16 h from initial injury. Nevertheless, prolonged fasciotomy did not increase the risk of amputation, and unsatisfactory functional results were more common, presenting as limited activity and chronic neurological symptoms of ankle and foot. Some authors support the concept of urgent revascularization within 6 h from the time at injury to repair could be predictive of poor functional outcome, particularly in these condition combined with prolonged warm ischemia and compartment syndrome [3, 9]. In fact, it may be related to decreased collateral circulation from superficial femoral artery due to osteofascial compartment hypertension [21]. Therefore, there was no better strategy for dealing with compartment syndrome other than early recognition and prompt decompression, especially in these patients with acute limb ischemia.

The results of the study also showed that the majority of patients with nerve dysfunction had significant lower ankle functional scores, which is in accordance with previous literature [22]. O’Malley et al. [23] found that patients with complete nerve injuries typically had a worse prognosis and appeared more severe ankle dysfunction than those with incomplete palsies. In addition, Woodmass et al. [24] identified vastly different prognosis between patients who suffered an incomplete common peroneal nerve palsy and complete common peroneal nerve palsy after knee dislocation in 13 studies with 214 patients. Eighty-seven percent of patients with an incomplete palsy had achieved a full motor recovery. By contrast, 38% of patients with a complete motor palsy had regained the ability to dorsiflex at the ankle. It is probably because complete common peroneal nerve palsy usually followed more severe soft tissue injuries and had concurrent motor end plate death [25, 26]. It has been reported that chronic pain may still exist for a long time even after corrective arthrodesis, peroneal or tibial nerve transfer, posterior tibial tendon transfer, which had the highest effect on functional outcomes [20, 27].

There is still no consensus regarding whether prolonged ischemia time contributed to amputation or poor function or not [3, 26]. Some authors support the concept of urgent revascularization within 6 h from the time at injury to repair could be predictive of poor functional outcome, particularly when the diagnosis is delayed [26]. The key of this concept is to decrease warm ischemia time. In our series, delayed flow-restored arterial repair was very common in all patients due to long transport or delay diagnosis. Nevertheless, our results showed duration of ischemia was not a risk factor for poor functional outcomes after surgery, which can be explained by compensatory collateral artery circulation from superficial femoral artery. It also needs to be noted collateral circulation blood supply will be affected under certain conditions, including compartment hypertension, complicated soft tissue injuries, occlusion of collateral artery pathways and unstable hemodynamics [19, 21, 28].

Although we improved treatments for concomitant injuries, better surgical techniques and multidisciplinary intervention, patients had a higher rate of surgical complications. In a retrospective study of 2175 patients in nationwide US inpatient database [29], patients with concomitant popliteal injury were also more likely to experience secondary complications, experience longer hospital stays and incur greater healthcare costs. The development of gastrocnemius necrosis after PAI has been described frequently in many studies due to severe trauma of gastrocnemius or no revascularization of the gastrocnemius artery [30, 31]. However, a surprising finding was that gastrocnemius necrosis was not the risk factor for poor ankle function in our series. This can be explained by the compensatory results of soleus and plantar flexor muscles and the patients' low demand for ankle function [32]. In fact, there is no existing evidence about whether gastrocnemius artery repair contributes to lower functional outcomes in these patients.

This study also has weaknesses. First is the small sample in size and the single-center design of the study. Second, we cannot thoroughly rule out some biases due to unknown and unmeasured confounding factors. Despite these limitations, the strengths of our study included the identification of a matched cohort to compare trauma-specific function of the salvaged limbs in patients after acute blunt popliteal artery injury, minimized confounding factor and long-term follow-up.

## Conclusion

Five-year trauma-specific outcomes showed that most patients who sustained blunt PAI had significant functional deficit associated with limited activity and chronic neurological symptoms of ankle and foot. Additionally, poor functional outcomes were related to nerve injury and compartment syndrome. Therefore, early and effective decompression for compartment syndrome remains the only potentially modifiable risk factor for improving functional outcomes following PAI.

## Data Availability

All the data will be available upon request to the corresponding author of the present paper.

## Abbreviations

PAI: Popliteal artery injury
ISS: Injury Severity Score
FIM: Functional Independence Measure
KSS: Knee Society Score
AHFS: Ankle–Hindfoot Scale
